# Supplementary material for: Biomolecular Corona Stability in Association with Plasma Cholesterol Level
Source: Nanomaterials (Basel). 2022 Aug 3;12(15):2661. doi: 10.3390/nano12152661 (PMC9370777; doi:10.3390/nano12152661)
Supplement: Supplementary file 1 [file nanomaterials-12-02661-s001.zip › nanomaterials-1813410-supplementary.pdf]

Supplementary Information

Biomolecular Corona Stability in Association with Plasma Cholesterol Level

Duong N. Trinh [1], Meda Radlinskaite [1,2], Jack Cheeseman [3,4], Gunter Kuhnle [5], Helen M. I. Osborn [4], Paula Meleady [6], Daniel I. R. Spencer [3] and Marco P. Monopoli [1,*]

1 Department of Chemistry, Royal College of Surgeons in Ireland, RCSI University of Medicine and Health Sciences, Dublin 2, Ireland; ngocduongtrinh@rcsi.ie
2 School of Physics & Clinical & Optometric Sciences, Technological University Dublin, Dublin 7, Ireland; c18307936@mytudublin.ie
3 Ludger Ltd., Culham Science Centre, Abingdon OX14 3EB, UK; jack.cheeseman@ludger.com (J.C.); daniel.spencer@ludger.com (D.I.R.S.)
4 School of Pharmacy, University of Reading, Whiteknights, Reading RG6 6AD, UK; h.m.i.osborn@reading.ac.uk
5 Department of Food and Nutritional Sciences, University of Reading, Whiteknights, Reading RG6 6AH, UK; g.g.kuhnle@reading.ac.uk
6 School of Biotechnology, Dublin City University, Dublin 9, Ireland; paula.meleady@dcu.ie
* Correspondence: marcomonopoli@rcsi.ie

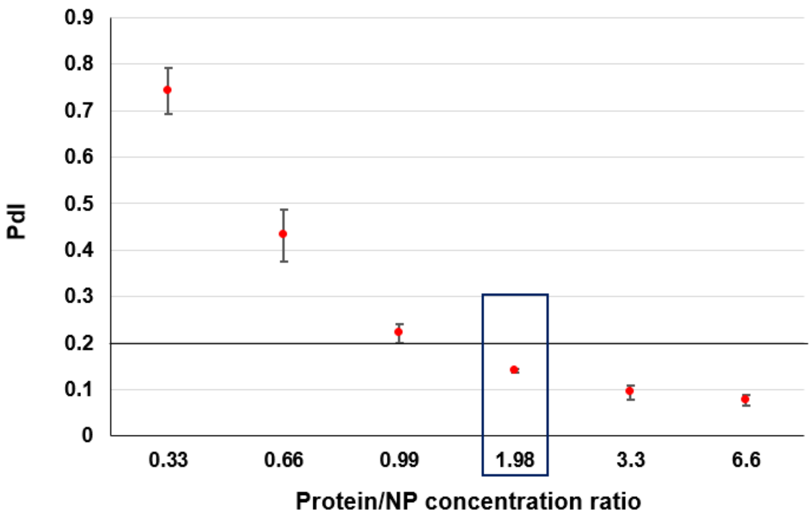

Figure S1. Silica coronas aggregated with decreasing plasma protein/NP concentration ratio. The protein/NP ratio of 1.98 was selected to obtain a corona with the PdI below 0.2. Error bar: SD of the mean (n = 3).

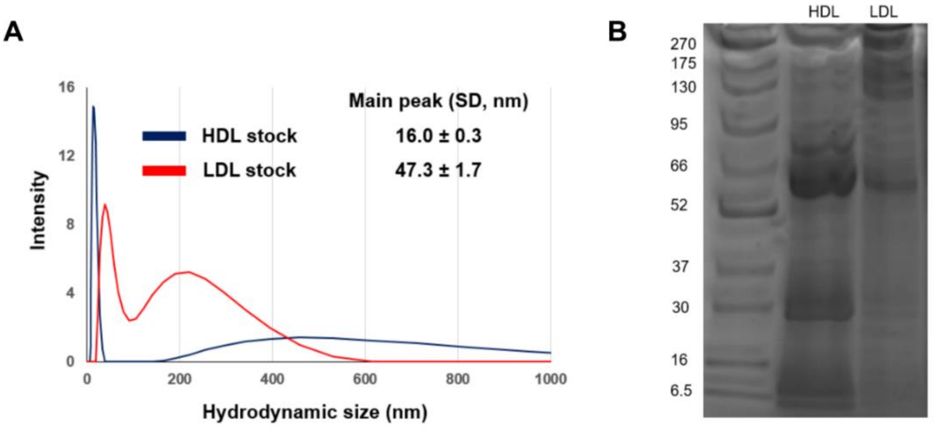

Figure S2. Plasma HDL and LDL characterization. (**A**) Hydrodynamic sizes of the HDL and LDL stock particles in PBS pH 7.4. (**B**) SDS-PAGE gel of the HDL and LDL.

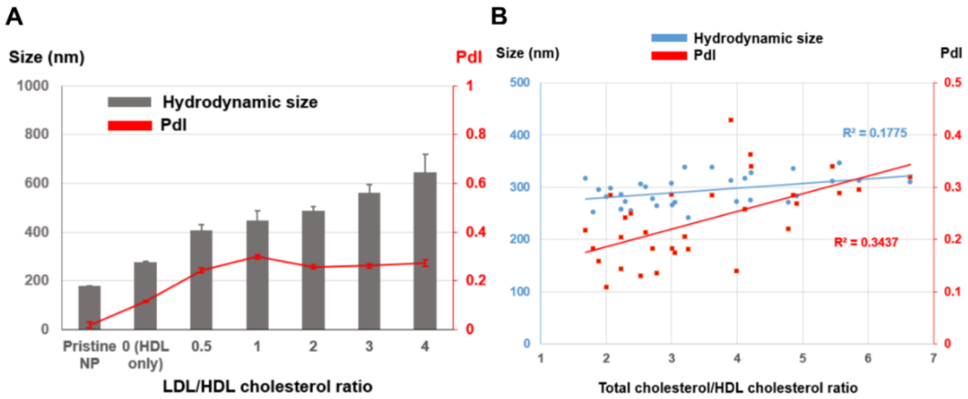

Figure S3. High LDL cholesterol induces polystyrene NP aggregation. (**A**) LDL induced polystyrene NPs; however, the aggregation was less severe than that of silica NPs. (**B**) Positive correlations between cholesterol ratio and both hydrodynamic size and PdI. The highest R^2 was observed between the ratio and sample PdI values (0.3437).

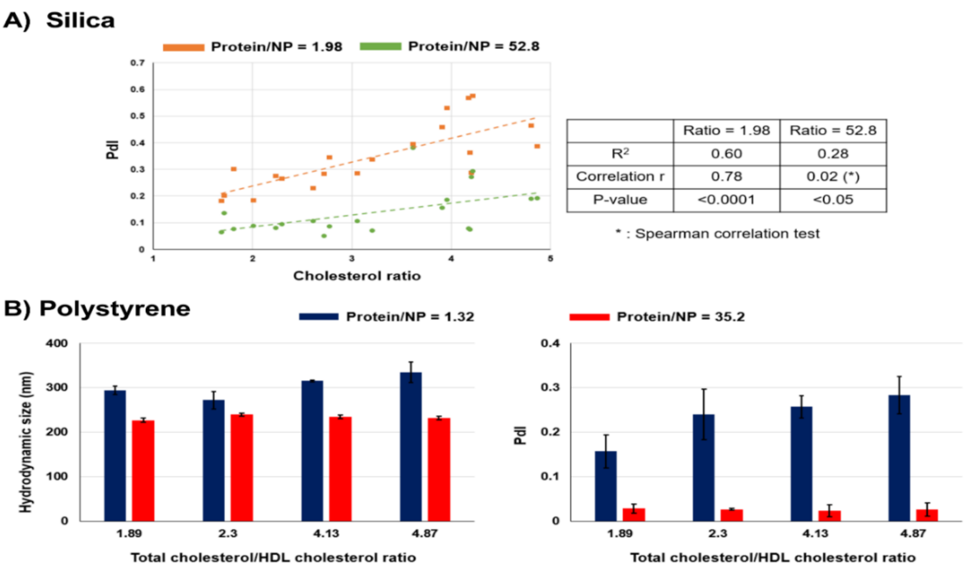

Figure S4. The colloidal stability variation was more noticeable with the corona formed at low protein/NP conditions. (**A**) A low correlation between cholesterol and PdI was present in the silica coronas at the protein/NP ratio of 52.8. (**B**) The hydrodynamic sizes and PdI of 200 nm polystyrene coronas formed in four different individuals' plasma samples at the ratio of 1.32 (blue) and 35.2 (red). The x-axis shows the plasma cholesterol ratios of the corresponding individual plasma samples. The coronas were stable even at very high plasma cholesterol level. The plasma samples used in this experiment were not the same as those in the cohort study. Error bar: SD of the mean (n = 3).

Table S1. DLS sizes of silica coronas obtained from pooled human plasma. The concentrations of the particles and plasma proteins were varied around three concentration ratios: 1.98; 26.4 and 52.8. The PdI values indicate good colloidal stability of all the samples.

Samples	Protein/NP ratio	Main peak (nm)	PdI
**1. Changing plasma concentration with fixed NPs' concentration**			
1 mg/mL NPs, 3% HP	1.98	164.8	0.062
1 mg/mL NPs, 40% HP	26.4	145.3	0.007
1 mg/mL NPs, 80% HP	52.8	129.5	0.006
**2. Changing NPs' concentration with fixed plasma concentration**			
40% HP, 0.5 mg/mL NPs	52.8	134.7	0.033
40% HP, 1 mg/mL NPs	26.4	145.3	0.007
40% HP, 13.3 mg/mL NPs	1.98	144.1	0.039
**3. Fixed plasma/NP concentration at 1.98**			
3% HP, 1 mg/mL NPs	1.98	164.8	0.062

40% HP, 13.3 mg/mL NPs	1.98	144.1	0.039
80% HP, 26.7 mg/mL NPs	1.98	160.1	0.089

Table S2. Top protein abundances of 100 nm silica and 200 nm carboxylated polystyrene plasma coronas. The coronas were formed in pooled plasma at the protein/NP ratios of 1.98 and 1.32, respectively. The abundances were ranked by the summed peptide intensity in binary logarithm form. While both of them shared fibrinogen at the top, apoA1 and apoB100 (bolded) were less abundant in the polystyrene corona.

Rank number	Silica corona		Polystyrene corona	
	Intensity (log_2)	Protein	Intensity (log_2)	Protein
1	37.3	**ApoA1**	35.4	Fibrinogen gamma chain
2	36.8	Fibrinogen gamma chain	34.7	Fibrinogen beta chain
3	36.8	Fibrinogen beta chain	34.6	Fibrinogen alpha chain
4	36.2	Fibrinogen alpha chain	33.1	Albumin
5	33.8	**ApoB100**	32.8	Complement C3
6	33.2	Histidine-rich glycoprotein	32.1	Complement factor H
7	32.6	Kininogen-1	31.4	Beta-2-glycoprotein 1
8	32.5	ApoE	31.4	**ApoA1**
9	32.1	ApoA4	30.9	Vitronectin
10	31.7	Fibronectin	30.9	Complement C4-B
11	31.5	Albumin	30.5	Fibronectin
12	31.4	Complement C3	30.4	**ApoB100**
13	31.1	Coagulation factor XII	29.9	Inter-alpha-trypsin inhibitor heavy chain H4

14	30.8	Complement C1q subcomponent subunit B	29.8	Histidine-rich gly-coprotein
15	30.6	Immunoglobulin heavy constant gamma 3	29.7	Kininogen-1

Table S3. Correlation coefficients (r) of the plasma cholesterol level (total cholesterol, HDL cholesterol and cholesterol ratio) and the polystyrene corona's colloidal stability (DLS size and PdI). Pearson correlation and Spearman correlation coefficients were calculated for normally distributed and non-normally distributed data, respectively. *: Pearson correlation coefficients. $P<0.05$ are bolded.

	**Total cholesterol**		**HDL cholesterol**		**Cholesterol ratio**	
	**r**	***P***	**r**	***P***	**r**	***P***
**Hydrodynamic size**	0.15 (*)	0.42	-0.26	0.163	0.42 (*)	**0.018**
**PdI**	0.31 (*)	0.09	-0.40	**0.026**	0.59 (*)	**0.001**
